# Dynamics of adipogenic promoter DNA methylation during clonal culture of human adipose stem cells to senescence

**DOI:** 10.1186/1471-2121-8-18

**Published:** 2007-05-29

**Authors:** Agate Noer, Andrew C Boquest, Philippe Collas

**Affiliations:** 1 Institute of Basic Medical Sciences, Faculty of Medicine, University of Oslo, PO Box 1112 Blindern, 0317 Oslo, Norway

## Abstract

**Background:**

Potential therapeutic use of mesenchymal stem cells (MSCs) is likely to require large-scale in vitro expansion of the cells before transplantation. MSCs from adipose tissue can be cultured extensively until senescence. However, little is known on the differentiation potential of adipose stem cells (ASCs) upon extended culture and on associated epigenetic alterations. We examined the adipogenic differentiation potential of clones of human ASCs in early passage culture and upon senescence, and determined whether senescence was associated with changes in adipogenic promoter DNA methylation.

**Results:**

ASC clones cultured to senescence display reduced adipogenic differentiation capacity in vitro, on the basis of limited lipogenesis and reduced transcriptional upregulation of *FABP4 *and *LPL*, two adipogenic genes, while *LEP *and *PPARG2 *transcription remains unaffected. In undifferentiated senescent cells, *PPARG2 *and *LPL *expression is unaltered, whereas *LEP *and *FABP4 *transcript levels are increased but not in all clones. Bisulfite sequencing analysis of DNA methylation reveals overall relative stability of *LEP*, *PPARG2*, *FABP4 *and *LPL *promoter CpG methylation during senescence and upon differentiation. Mosaicism in methylation profiles is maintained between and within ASC clones, and any CpG-specific methylation change detected does not necessarily relate to differentiation potential. One exception to this contention is CpG No. 21 in the *LEP *promoter, whose senescence-related methylation may impair upregulation of the gene upon adipogenic stimulation.

**Conclusion:**

Senescent ASCs display reduced in vitro differentiation ability and transcriptional activation of adipogenic genes upon differentiation induction. These restrictions, however, cannot in general be attributed to specific changes in DNA methylation at adipogenic promoters. There also seems to be a correlation between CpGs that are hypomethylated and important transcription factor binding sites.

## Background

Mesenchymal stem cells (MSCs) have been identified in many human tissues and are thought to be responsible for tissue homeostasis. Adipose tissue constitutes an abundant source of MSCs [1,2]. We have shown that adipose stem cells (ASCs) with a CD45^-^CD31^-^CD34^+^CD105^+ ^phenotype can be isolated with ~99% purity from the stromal vascular fraction of human liposuction material [3]. Human ASCs exhibit primarily mesodermal differentiation capacity in vitro [1], and in vivo may contribute to improvements of osteogenic [4], neuronal [5], immune [6] and vascular functions [7]. ASCs display an immunophenotype similar to bone marrow MSCs and express genes extending across the three germ layers, supporting a differentiation potential toward non-mesodermal lineages [3,8,9].

Single cell-derived clonal cultures of human ASCs, however, show that differentiation capacity in vitro varies between cell clones [3]. Analysis of DNA methylation at promoters of lineage-specific genes showed that freshly isolated, undifferentiated ASCs display mosaically under-methylated adipogenic gene promoters (*LEP*, *PPARG2*, *FABP4*, *LPL*); in contrast, myogenic (*MYOG*) and endothelial (*CD31*, *CD144*) promoters are hypermethylated [10,11]. Methylation of a cytosine in a CpG dinucleotide is a heritable modification that favors genomic integrity, ensures proper regulation of gene expression and is essential for long-term gene silencing [12]. Methylation profiles are maintained in clonal ASC cultures up to 4 passages, or 20 population doublings from single cells, and do not correlate with gene expression level in undifferentiated or differentiated cells [10]. The unmethylated state of adipogenic promoters, together with the hypermethylation of endothelial and myogenic promoters and the maintenance of these methylation patterns after adipogenic, osteogenic or endothelial differentiation [10,11,13] suggest that ASCs are epigenetically committed to adipogenic differentiation preferentially over other lineages.

Human and murine MSCs cultured in vitro undergo replicative senescence and exhibit reduced differentiation potential upon prolonged culture [14-19]. Loss of functionality of senescent MSCs is presumably due to accumulation of oxidative damage on DNA and proteins [20,21] and to telomerase dysfunction [22]. In vivo, reduced ability of MSCs to contribute to tissue repair in aged individuals has been attributed to a loss of MSC function rather than to diminished stem cell numbers [23].

Aging is accompanied by changes in DNA methylation in tumor suppressor genes [24] and by stochastic methylation events throughout the genome [25]. Culture may also induce random DNA methylation errors as a result of infidelity in maintenance DNA methylation [26,27]. In this study, we investigate the extent to which senescent clonal cultures of human ASCs exhibit reduced differentiation potential in vitro and whether this may be attributed to changes in promoter DNA methylation.

## Results

### Senescent ASCs display reduced adipogenic differentiation potential

**Figure 1:**
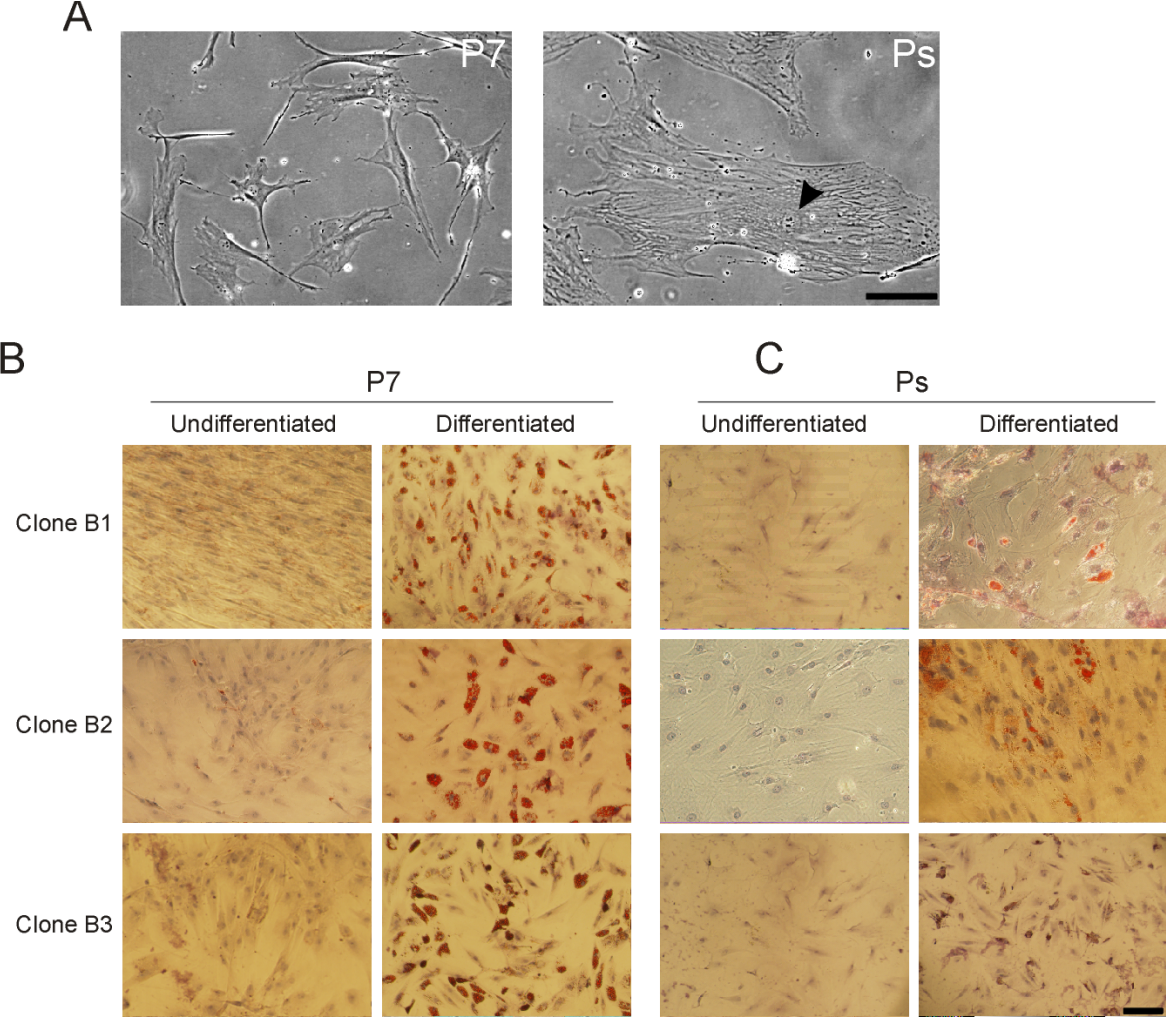
Adipogenic differentiation of human ASC clones in early and late passage. (A) Morphology of an ASC clone (B2) at P7 and at senescence (Ps). Arrow points to the nucleus of a large and flattened single cell at Ps. These phenotypes are typical for early passage and senescent human ASCs cultured clonally or as polyclones. (B) Three ASC clones (B1, B2, B3) were induced to differentiate toward the adipogenic pathway for three weeks, starting at P7 (B) or Ps (C). Ps corresponded to P18 (clone B1), P16 (clone B2) and P17 (clone B3). Cells were stained with Oil Red-O to visualize lipid droplets and with hematoxylin to visualize nuclei. Bars, 100 μm.

To investigate the effect of senescence on differentiation ability of ASCs, we derived clonal cultures of ASCs from single freshly isolated cells [3]. The adipogenic differentiation capacity of these clones in early passage cultures (passage 4; P4) has been described previously [10]. Three clones, designated B1, B2 and B3, were cultured to senescence. Senescence was reached at passage P18 (i.e., after 48 population doublings) for clone B1, P16 (44 population doublings) for clone B2 and P17 (46 population doublings) for clone B3. Senescence was manifested by cell cycle arrest, as judged by the absence of cell division on the basis of cell counts upon at least two rounds of subculture after these passage numbers were reached (data not shown), and by typical enlarged cell morphology (Fig. 1A). Passage number achieved at senescence is referred to as Ps hereafter.

To evaluate the differentiation capacity of early passage vs. senescent cells, all clones were induced to differentiate toward the adipogenic pathway for three weeks, starting either at P7 or at Ps. All P7 clones differentiated efficiently, on the basis of Oil Red-O staining (Fig. 1B). Differentiation, however, was impaired in senescent cells (Fig. 1C, Ps). Note that differentiation was elicited for each clone with 20,000 cells/cm^2 ^both at P7 and at Ps, so we could not attribute differences in the extent of adipogenesis to differences in cell density.

**Figure 2:**
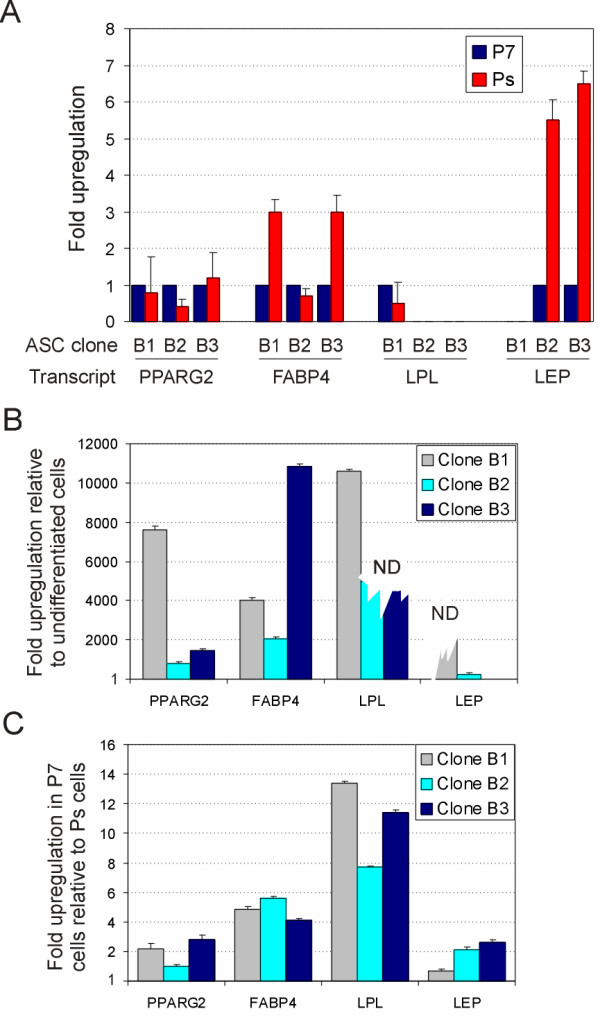
Expression of adipogenic genes upon differentiation of P7 and senescent ASCs. (A) *LEP*, *PPARG2*, *FABP4 *and *LPL *transcript levels were determined in undifferentiated Ps cells (clones B1, B2, B3) relative to undifferentiated P7 cells. For each clone, the mRNA level detected at P7 was attributed a value of 1 and the mRNA level in the same clone at Ps was expressed relative to that. A value of 0 (no bar) indicates that the gene is not expressed. (B) At senescence, each clone was induced to differentiate and mRNA levels compared to those of undifferentiated cells (level 1). 'ND' indicates that the gene was upregulated but relative levels could not be determined because transcripts were undetected in undifferentiated cells (jagged bars). (C) Transcript levels in cells differentiated at P7 were expressed relative to those in cells differentiated at Ps (level 1). Each panel shows the mean fold upregulation from triplicate RT-PCRs from at least two separate cDNA preparations.

Quantitative analysis of expression of adipogenic genes in undifferentiated P7 and Ps cells by real time reverse transcription (RT) polymerase chain reaction (PCR) corroborated the reduced ability of senescent ASCs to commit to adipogenesis. In undifferentiated P7 cells, expression of peroxisome proliferator-activated receptor gamma 2 (*PPARG2*) occurred at low levels and was not significantly altered in senescent cells in any of the clones (*P *> 0.05; Fig. 2A). Expression of fatty acid-binding protein 4 (*FABP4*) [28] mRNA, also detected at low levels at P7, was unaltered at Ps in clone B2 (*P *> 0.1), but was upregulated 3-fold in clones B1 and B3 (*P *< 0.01 for each clone, relative to undifferentiated cells; Fig. 2A). Transcripts for lipoprotein lipase (*LPL*) were undetectable at P7 or Ps in clones B2 and B3, consistent with previous observations [10], but were expressed at a low level in both P7 and senescent cells in clone B1 (Fig. 2A). Lastly, leptin (*LEP*) mRNA was undetected in clone B1 but was upregulated 5- to 6-fold at senescence in clones B2 and B3 (*P *< 0.01 relative to undifferentiated cells; Fig. 2A). It is noteworthy however, that all transcripts detected in undifferentiated P7 ASCs were expressed at very low levels, as illustrated by elevated Ct values (> 35) despite high real time PCR efficiency (data not shown).

Three weeks of adipogenic stimulation initiated at Ps promoted upregulation of all genes relative to undifferentiated cells in all clones (*P *< 0.01 for any gene examined), except for *LEP *which remained unchanged in clone B3 (*P *> 0.1; Fig. 2B). Extent of gene upregulation varied between clones, reflecting differences in the ability of each clone to commit to adipogenesis even in late passage culture (*P *< 0.01 between clones, for each gene). This is similar to what we previously observed with the same clones at passage 4 [10]. Note, however, that a significant part of this variation was due to the low levels of transcripts detected in undifferentiated cells, small variations in which can result in large differences in relative upregulation upon differentiation (small variations in PCR efficiency also affect quantification of mRNA levels [29]). Although transcriptional activation took place, *LPL *mRNA levels in clones B2 and B3, and *LEP *mRNA level in clone B3 could not be quantified as these genes were not expressed in undifferentiated cells (see Fig. 2A). Furthermore, *FABP4 *and *LPL *mRNA levels in ASCs after three weeks of adipogenic stimulation at P7 were higher than after induction of differentiation at Ps (*P *< 0.05 for *FABP4 *and *P *< 0.005 for *LPL*; one-sample *t*-test), while those of *PPARG2 *and *LEP *were not different (*P *> 0.05, one-sample *t*-test; Fig. 2C). Impaired lipid accumulation and transcriptional activation of adipogenic genes during culture of ASCs to senescence illustrate a reduced commitment to adipogenesis, despite stable or moderately upregulated transcript levels in undifferentiated cells.

### Adipogenic promoter DNA methylation profiles remain stable upon culture to senescence

**Figure 3:**
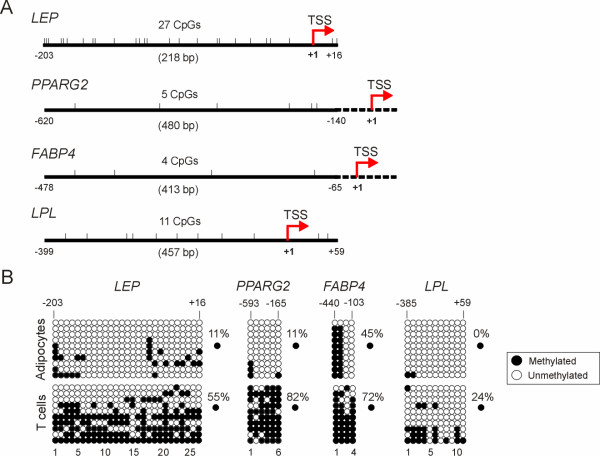
DNA methylation analysis of adipogenic promoters in human adipocytes and T cells. (A) Map of CpG dinucleotides (tick marks) examined in the *LEP*, *PPARG2*, *FABP4 *and *LPL *promoters. Regions examined are indicated by the continuous line. Numbers are relative to the transcription start site (TSS). (B) Bisulfite sequencing of *LEP*, *PPARG2*, *FABP4 *and *LPL *promoters in differentiated human SGBS adipocytes and in purified peripheral blood T cells. Ten bacterial clones of PCR products were sequenced. Each row represents one bacterial clone with one circle symbolizing one CpG. Top numbers indicate position of CpGs relative to the TSS. Bottom numbers are CpG numbers, with No. 1 being the 5'-most cytosine in the sequence examined.

To determine whether reduced transcriptional level of adipogenic genes in senescent ASCs had an underlying epigenetic basis, we assessed the DNA methylation status in the *LEP*, *PPARG2*, *FABP4 *and *LPL *promoters by bisulfite sequencing. Bisulfite conversion of DNA relies on the deamination of unmethylated cytosines into uracils, while methylated cytosines are protected from deamination. PCR converts uracils to thymidines and sequencing of several PCR products cloned in bacteria enables a quantitative assessment of the position and number of methylated cytosines in the region examined [30]. Regions analyzed by bisulfite sequencing in the *LEP*, *PPARG2*, *FABP4 *and *LPL *promoters are shown in Fig. 3A. We first validated the significance of the regions examined by showing that the extent of methylation was consistently lower in all promoters in differentiated SGBS (Simpson-Golabi-Behmel Syndrome) human adipocytes (0–45% methylated CpGs) than in peripheral blood T cells (24–72% methylation; Fig. 3B; *P *< 10^-4 ^for each promoter; *t*-test).

**Figure 4:**
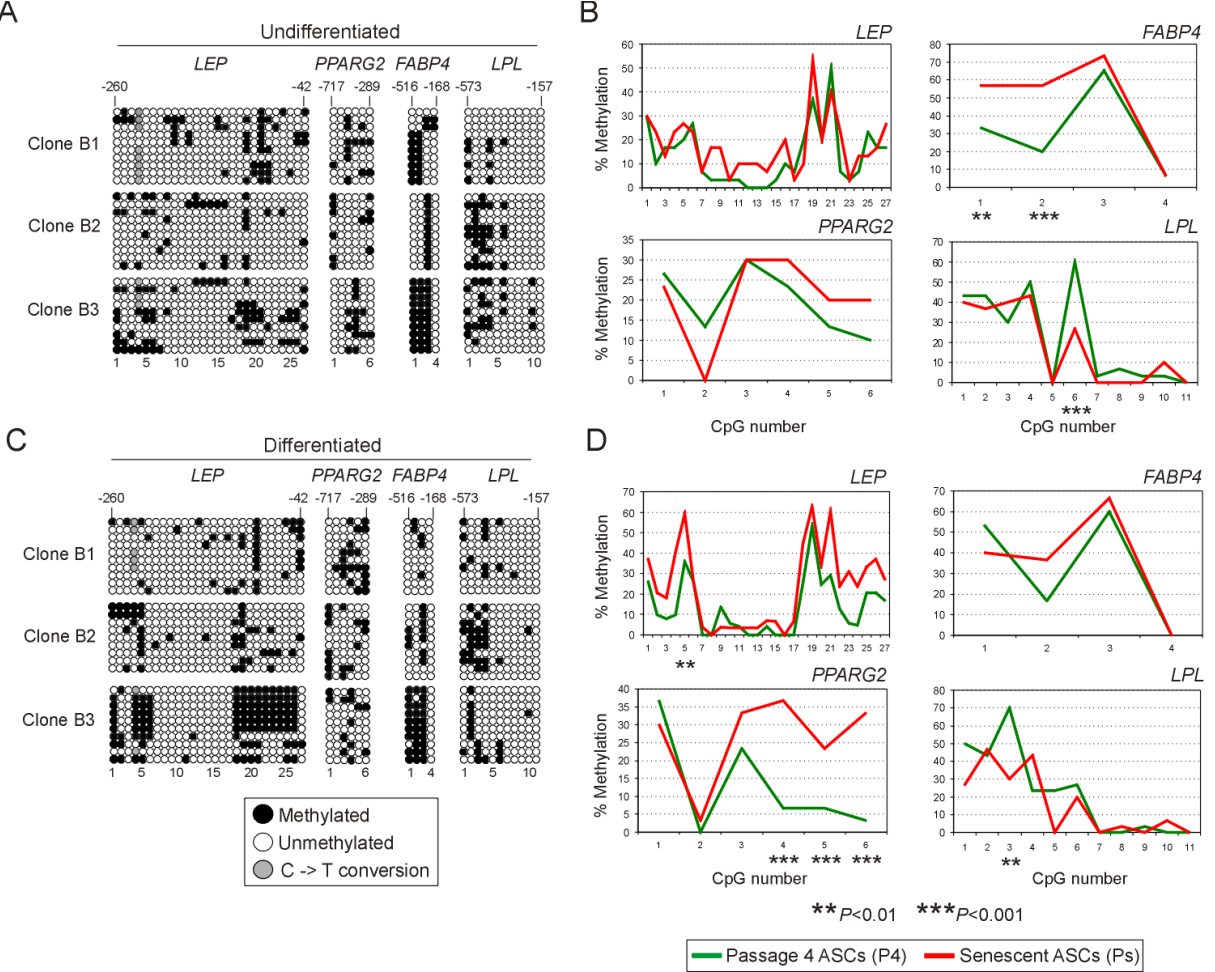
DNA methylation analysis of adipogenic promoters in undifferentiated and differentiated ASCs at passage 4 (P4) and senescence (Ps). (A) Bisulfite sequencing of *LEP*, *PPARG2*, *FABP4 *and *LPL *in undifferentiated Ps cells. Numbers are as in Figure 3. (B) Percentage of individual methylated CpGs (● in A) in undifferentiated P4 (green) and Ps (red) cells (average of clones B1, B2, B3). Data for individual undifferentiated ASC clones are shown in Additional file 1, Supplementary Figure. (C) Bisulfite sequencing of Ps cells after adipogenic differentiation. Numbers are as in Figure 3. (D) Percentage of individual methylated CpGs (● in C) in differentiated cells at Ps. Data for individual differentiated clones are shown in Additional file 1, Supplementary Figure 2. **P < 0.01, ***P < 0.001 (*t*-tests).

DNA methylation profiles of undifferentiated senescent cells are shown in Fig. 4A and illustrated for all individual clones in Additional file 1, Supplementary Fig. 1. The *LEP, PPARG2 *and *LPL *promoters were largely hypomethylated in all clones examined, with percentages of methylation ranging from 7 to 29% (Table 1, *Undifferentiated*). We found that the preferred areas of methylation in each locus we previously identified in early passage (P4) cells [10] were maintained in Ps cells (Fig. 4A). Furthermore, as in P4 cells, methylation profiles were mosaic in senescent cells, both between ASC clones and within a clonal population (Fig. 4A). The *FABP4 *promoter displayed greater variation in global methylation with clone B2 being 25% methylated, while B3 was 75% methylated (Fig. 4A; Table 1, *Undifferentiated*; Additional file 1, Supplementary Fig. 1C). Note that although adipogenic differentiation potential was evaluated in ASCs at P7 in this study, early passage methylation status was previously determined at P4 [10]; however no or minimal methylation differences were anticipated between P4 and P7, relative to senescence (P16-18).

**Table 1:** Percentage of CpG methylation in the *LEP*, *PPARG2*, *FABP4 *and *LPL *promoters in undifferentiated and differentiated ASC clones at passage 4 (P4) and at senescence (Ps)

		Percent methylated cytosines in promoter region examined							
		
Gene		*LEP*		*PPARG2*		*FABP4*		*LPL*	
Passage No.		P4	Ps	P4	Ps	P4	Ps	P4	Ps

*Undifferentiated*									
ASC clone	B1	9	17^a^	10	20^c^	15	45^a^	20	8^a^
	B2	7	14^a^	15	18^ns^	30	25^ns^	27	26^ns^
	B3	24	24^ns^	29	23^ns^	50	75^a^	19	19^ns^

*Differentiated*									
ASC clone	B1	13	13^ns^	5	33^a^	12	13^ns^	17	11^ns^
	B2	4	17^a^	17	25^c^	22	25^ns^	29	24^ns^
	B3	22	38^a^	17	22^ns^	50	70^b^	18	14^ns^

Chi-square tests: ^a ^*P *< 0.001; ^b ^*P *< 0.005; ^c ^*P *< 0.05; ^ns ^non-significant.

Methylation profiles of each promoter in senescent cells (Fig. 4B, red lines) were compared to those obtained earlier [10] in the same ASC clones cultured to P4 (Fig. 4B, green lines). Methylation profiles remained overall similar during culture to senescence (Fig. 4A, B). However, two CpGs were found to be hypermethylated in *FAPB4 *(CpGs 1 and 2; *P *< 0.01 and *P *< 0.001, respectively; *t*-tests), owing to hypermethylation in clones B1 and B3 (Additional file 1, Supplementary Fig. 1C). Moreover, we found one CpG to be hypomethylated in *LPL *(CpG 6; *P *< 0.001; *t*-test; Fig. 4B) also owing to demethylation of this cytosine in clones B1 and B3 (Additional file 1, Supplementary Fig. 1D). When global percentages of methylation were compared between undifferentiated individual ASC clones at P4 and Ps, however, *LEP*, *PPARG2 *and *FABP4 *were found to be hypermethylated upon senescence in clone B1, while clones B2 and B3 only showed *LEP *and *FAPB4 *hypermethylation, respectively (Table 1, *Undifferentiated*). *LPL *promoter methylation was reduced between P4 and Ps in clone B1 and remained stable in the other clones (Table 1, *Undifferentiated*).

These results indicate that overall, adipogenic promoters retain the largely hypomethylated profile that characterizes ASCs. Nevertheless, a few CpGs become hyper- or hypomethylated upon senescence in the *FABP4 *and *LPL *promoters, respectively, albeit not in all clones.

### Adipogenic differentiation maintains methylation profiles at adipogenic promoters in senescent ASCs

**Figure 5:**
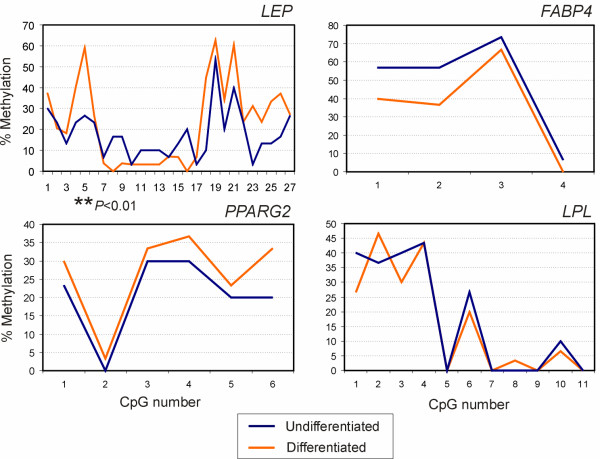
DNA methylation profiles of adipogenic promoters in undifferentiated and in adipogenic stimulated senescent ASCs. Data were obtained by bisulfite sequencing as shown in Figure 4. Percentages of methylated CpGs are shown (average of clones B1, B2 and B3). **P < 0.01 (*t*-test).

We next examined the methylation profile of adipogenic promoters after adipogenic stimulation in senescent cells and compared these profiles to those of cells differentiated at P4 [10]. Methylation patterns were similar in the *LEP *promoter in differentiated P4 and Ps cells (Fig. 4C, D; Table 1, *Differentiated*), with only two CpGs, No. 5 and 21, being hypermethylated at Ps (*P *< 0.01; *t*-tests; Fig. 4D). Upon examination of individual clones, however, clones B2 and B3 displayed enhanced methylation at senescence (*P *< 0.001; *t*-tests; Table 1, *Differentiated*) due to hypermethylation of several CpGs (Additional file 1, Supplementary Fig. 2A). In *PPARG2*, enhanced methylation was seen at Ps compared to P4 (Fig. 4C, D; Table 1, *Differentiated*; *P *= 0.011; *t*-test) due to hypermethylation of CpGs No. 4, 5 and 6 (Fig. 4D; *P *< 0.001 for each cytosine; *t*-tests; Table 1; Additional file 1, Supplementary Fig. 2B). The *FABP4 *and *LPL *promoters displayed remarkably similar methylation profiles in Ps and P4 differentiated cells (Fig. 4C, D), with only clone B3 showing *FABP4 *hypermethylation in differentiated Ps cells (Table 1) due to enhanced methylation of CpG 2 (Additional file 1, Supplementary Fig. 2C). Finally, we found that methylation profiles of all promoters were overall similar in undifferentiated and in adipogenic-stimulated Ps cells (Fig. 5, showing a direct comparison of data of Figs. 4B and 4D, red curves), except for hypermethylation of CpG 5 in the *LEP *promoter (Fig. 5; *P *< 0.01; *t*-test). We concluded that overall, methylation patterns in adipogenic promoters are similar between adipogenic-stimulated cells in early passage and at senescence. However, as with undifferentiated cells, specific CpGs appear to be hyper- or hypomethylated in a promoter-specific and cell clone-specific manner.

We occasionally noted the substitution of a cytosine for a thymidine (C:T substitutions) in some *LEP *bisulfite sequences, consistently at CpG No. 4 (Fig. 4A, C, blue marks). Sequences were unambiguous and displayed either a methylated C or an unmethylated C (the latter read as A, as shown in Additional file 1, Supplementary Fig. 3A) or, unexpectedly a T (read as a T; Additional file 1, Supplementary Fig. 3B). To account for this substitution, it has been proposed that CpG methylation increases the mutation rate from CpG to TpG or CpA, as suggested by the lower number of CpGs observed over that expected in mammalian genomes [31,32].

In summary, methylation profiles at adipogenic promoters are generally maintained upon clonal culture of ASCs to senescence. Nevertheless, at the level of individual clones, methylation may be differentially affected in a gene-independent manner, but this does not necessarily correlate with transcriptional changes. Adipogenic stimulation of early passage or senescent cells does not markedly affect adipogenic promoter DNA methylation.

## Discussion

### Reduced adipogenic differentiation potential of senescent ASCs is not related to changes in promoter DNA methylation

Little is known on alterations in DNA methylation in MSCs during in vitro aging and how these may relate to changes in differentiation potential. We examined three clonal lines of ASCs with an ability to differentiate toward adipogenic [10], osteogenic and endothelial pathways [11,13]. Similarly to cultured bone marrow MSCs [20,33], all ASC clones cultured to senescence displayed reduced adipogenic differentiation potential in vitro, on the basis of lipid synthesis and transcriptional upregulation of, primarily, *FABP4 *and *LPL *relative to the same clones in early passage cultures. Interestingly however, basal transcription of these genes was unaltered (*PPARG2*, *LPL*) or moderately enhanced (*FABP4*, *LEP*) in undifferentiated cells at Ps. Thus, reduced transcription efficiency in differentiated senescent cells cannot be attributed to lower transcript levels in the undifferentiated state.

What may be the basis for reduced adipogenic potential in senescent ASCs? First, *PPARG2*, a master regulator of adipogenesis [34], is upregulated to a level similar in early passage and senescent cells, thus we cannot explain reduced adipogenesis by PPARγ insufficiency. Second, adipogenic stimulation of senescent cells upregulates *LPL *expression, albeit to a lower level than in early passage cells. A suboptimal transcription level is likely to affect translation, preventing further phenotypic changes. Of note, transcription without detectable protein expression seems to be a feature of undifferentiated ASCs [3]. Third, transcription or synthesis of transcriptional regulators of adipogenic genes, such as C/EBPα [34], BMP4 [35] or ADD1/SREBP1c [36] may be compromised in senescent cells and may account for adipogenesis deficiency despite the putative availability of binding sites (see below). Indeed, failure to express C/EPBα results in inability to develop white adipose tissue in vivo [37,38]. Fourth, adipogenic failure may also be due to impaired downregulation of GATA2/3, pre-adipocyte factor 1 or Wnt10b, whose expression blocks cells in a pre-adipocyte stage [39].

To determine whether there was an epigenetic basis for reduced differentiation potential in senescent cells, we examined the DNA methylation profiles in the *LEP*, *PPARG2*, *FABP4 *and *LPL *promoters. Senescent cells maintained the overall mosaic hypomethylation of *LEP*, *PPARG2 *and *LPL *previously reported in P4 cells [10]. *FABP4 *exhibited hypermethylation in two undifferentiated ASC clones (B1, B3) at senescence. However, this did not correlate with reduced transcription potential upon adipogenic stimulation because clone B2, which is not hypermethylated at senescence, showed similar a reduction in transcription level to clones B1 and B3. Mosaic methylation has been reported in other primary cell cultures [26,27,40] and is likely the result of stochastic errors in maintenance methylation which occur over many rounds of replication. On the basis of our observations, therefore, it is difficult to attribute any change in promoter methylation to an effect of senescence per se.

### Relationship between CpG methylation and binding of transcriptional regulators

Identification of relationships between the most hypomethylated areas in adipogenic promoters and binding sites for transcription factors argues that most, but not all, these sites remain available during culture. In the *LEP *promoter, methylation of CpGs No. 4–6 in clones B2 and B3 covers a recognition site for the methyl-CpG binding protein Kaiso [41,42]. Moreover, two Sp1binding sites span CpGs No. 15 and 16 while CpG No. 17 lies in a site occupied by an unidentified factor [43]. CpGs No. 15–17 are unmethylated at P4 and at senescence, presumably leaving these binding sites available. Additionally, CpG No. 21 lies within a C/EBPα recognition site flanked by E boxes for putative ADD1/SREBP1 binding, all of which are important for *LEP *expression [43,44]. In contrast to CpGs No.15–17, CpG No. 21 is hypermethylated in senescent ASCs, but only in the context of adipogenic stimulation, in clones B1 and B3. CpG No. 21 hypermethylation may account for limited or undetectable *LEP *upregulation in adipogenic stimulated senescent cells. Moreover, we found that *LEP *transcripts increased 5–6-fold in undifferentiated senescent clones B2 and B3, but not in clone B1, which displayed heaviest methylation on CpG No.21. Therefore, methylation of CpG No.21 correlates with impaired *LEP *transcriptional upregulation in senescent cells in the context of adipogenic stimulation.

Methylation profiles of the *LPL *and *FABP4 *promoters further illustrates a functional relationship between DNA methylation and transcription factor binding. The *LPL *promoter contains a PPAR responsive element between CpGs No. 7 and 8 [45,46], a sterol-responsive element acting as binding site for SREBP1c [36], and between CpGs No. 9 and 10, a CAAT box and a binding sequence for Oct1 and TFIIB. Notably, CpGs No. 7–11 are consistently unmethylated in both early and late passage ASCs, thereby enabling transcriptional activation of *LPL *upon differentiation even in senescent cells. Availability of transcription factor binding sites in senescent ASCs also yields for the *FABP4 *promoter, which is strongly activated upon adipogenic stimulation. Notably, this promoter harbors a C/EBPα binding site close to CpG No. 4 [47], the only cytosine unmethylated regardless of ASC donor [10], cell clone and differentiation state in early passage and in senescent cells.

### Promoter DNA methylation during senescence and aging

Stochastic methylation in senescing ASCs is reminiscent of random methylation patterns established in human intestinal crypt stem cells in aging individuals [48,49]. Aging-related random methylation has been proposed to occur as a result of failure of maintenance methyltransferase activity, and/or exposure to environmental factors and health of the individual [24,50,51]. Our results provide evidence that methylation changes detected in senescent ASCs do not correlate with transcriptional defects and reduced differentiation potential. Likewise, there is to date little evidence linking DNA methylation of lineage-specific genes and aging-related impairment of stem cell function (tissue homeostasis) [20], stochastic changes in gene expression [52] and genome integrity [53]. Nevertheless, comparing aging to in vitro replicative senescence should be made with caution, as age of MSCs does not always affect differentiation potential in vitro [54].

In summary, our results argue that reduction of adipogenic potential in senescent ASCs may relate to transcriptional defects upon adipogenic stimulation. However, it cannot in general be attributed to specific methylation changes in adipogenic promoters, and thereby to availability of transcription factor binding sites regulated by CpG methylation. One exception to this contention is one CpG in the *LEP *promoter (CpG No. 21 in the present study), whose senescence-related methylation may impair upregulation of the gene upon adipogenic stimulation. There also seems to be a correlation between regions of CpGs hypomethylation and important transcription factor binding sites.

## Conclusion

ASCs cultured to senescence display reduced in vitro differentiation ability and transcriptional activation of adipogenic genes. These restrictions, however, cannot be attributed to specific changes in DNA methylation on adipogenic promoters. There also seems to be a correlation between CpGs that are hypomethylated and important transcription factor binding sites.

## Methods

### Adipose stem cell isolation and clonal culture

Cells with a CD45^-^CD31^-^CD34^+^CD105^+ ^phenotype (ASCs) were isolated from the stromal vascular fraction of human adipose tissue as described [3]. In short, lipoaspirates obtained from the hip or thigh regions of female donors below 40 years of age were digested with collagenase and DNase I, and adipocytes were separated from stromal vascular cells by centrifugation. Stromal cells were strained through sieves and CD45^- ^cells were retrieved by negative selection. CD31^- ^cells were in turn negatively selected using FITC-conjugated anti-human CD31 antibodies and anti-FITC microbeads. Isolated ASCs are CD34^+^CD105^+^so no negative selection was performed for these markers [3]. For clonal expansion, single freshly isolated ASCs were cultured in 48-well plates in DMEM/F12 medium containing 50% fetal bovine serum. After ~16 h, the medium was replaced with DMEM/F12/10% fetal bovine serum and after 3 weeks, colonies were passaged by trypsinization. The three ASC clones used in this study (clones B1, B2, B3) have been previously characterized for their ability to differentiate toward adipogenic, osteogenic and endothelial cell lineages [10,11,13].

### Adipogenic differentiation

Cells were cultured to confluency in DMEM/F12/10% serum and stimulated for 3 weeks with 0.5 mM 1-methyl-3 isobutylxanthine, 1 μM dexamethasone, 10 μg/ml insulin (Novo Nordisk) and 200 μM indomethacin (Dumex-Alpharma). To visualize lipid droplets fixed cells were stained with Oil Red-O as described earlier [3].

### Quantitative RT-PCR

Total RNA was purified using the DNAse-containing RNeasy Micro Kit (Qiagen). RT-PCR was carried out from 500 ng total RNA using the Iscript cDNA synthesis kit (BioRad). Quantitative RT-PCR reactions were performed in triplicates from two separate cDNA preparations on a MyiQ Real-time PCR Detection System using IQ SYBR^® ^Green (BioRad). RT-PCR primers used have been published earlier [10]. SYBR^® ^Green PCR conditions were 95°C for 4.5 min and 40 cycles of 95°C 30 sec, 60°C 30 sec and 72°C 30 sec, and transcript levels calculated [29] using *GAPDH *as a normalization control [10]. RT-blank control PCRs showed no product amplification for all genes examined in this study (data not shown). Relative transcripts levels were compared using paired *t*-tests and statistical significance expressed as a two-tailed *P *value with *P *< 0.05 considered as significant. When relevant, transcript levels were compared to a "reference" level (level 1) using one-sample *t*-tests.

### Bisulfite sequencing

Methylation of four adipogenic gene promoters was analyzed by bisulfite sequencing. The *LEP *promoter region examined, and known to be regulated by methylation [44], covered nucleotides 2719–2937 (GenBank U43589) and spanned 27 potentially methylated cytosines within nucleotides -203 to +16 with respect to the transcription start site (TSS). The *PPARG2 *promoter region [55] spanned nucleotides 108–587 (GenBank AB005520) and included 6 CpGs within nucleotides -620 to – 140 with respect to the TSS. The *FABP4 *(GenBank NM_001442) promoter region encompassed 4 CpGs within nucleotides -478 to -65 with respect to the TSS. The *LPL *promoter region (GenBank X68111) spanned bases 1321–1777 and included 11 CpGs within nucleotides -398 to +59 with respect to the TSS. Sequence positioning was identified using Ensembl [56].

For bisulfite conversion, DNA was purified by phenol-chloroform-isoamylalcohol extraction. Cells were lysed for 10 min in lysis buffer (10 mM Tris-HCl, pH 8, 100 mM EDTA, 0.5% SDS) and digested with 0.1 mg/ml Proteinase K overnight. Bisulfite conversion was performed using the MethylEasy DNA Bisulfite Modification kit as detailed by the manufacturer (Human Genetic Signatures) [10]. Conversion rates, assessed by conversion of all cytosines in non-CpG dinucleotides to uracils (reading as thymidines after PCR), was 100% (data not shown). Converted DNA was used fresh or stored at -20°C. Converted DNA was amplified by PCR using primers commercially available (Human Genetic Signatures) for *LEP*. Primers for *PPARG2*, *FABP4 *and *LPL *were designed using Methprimer [57] and previously reported [10]. For *PPARG2*, *FABP4 *and *LPL*, PCR conditions were 95°C for 7 min and 40 cycles of 95°C 1 min, 54°C 2 min and 72°C 2 min, followed by 10 min at 72°C. For *LEP*, nested PCRs were performed, each with 95°C for 3 min and 30 cycles of 95°C 1 min, 50°C 2 min and 72°C 2 min, followed by 10 min at 72°C. PCR products were cloned into *E. coli *by TOPO TA cloning (Invitrogen) and reverse-sequenced (MWG Biotech).

Methylation data are represented as filled circles (methylated CpG) and empty circles (unmethylated CpG) for each bacterial clone obtained. Each row of circles represents methylation pattern based on the sequence of one cloned PCR product (i.e., one genomic allele). Global methylation percentages for a given promoter were compared using paired *t*-tests. Numbers of methylated cytosines for a given CpG were compared between cell populations using paired *t*-tests and significance was expressed using two-tailed *P *values.

## Abbreviations

ASC, adipose stem cells; MSC, mesenchymal stem cell; PCR, polymerase chain reaction, TSS, transcription start site

## Authors' contributions

AN: experimental design, all practical work, data analysis, figures, writing. ACB: derivation of ASC clones, differentiation. PC: experimental design, figures, supervision, writing. All authors read and approved the final manuscript.

## Supplementary Material

Additional file 1 Supplementary Figures with legends. The data provided show DNA methylation profiles for adipogenic gene promoters in undifferentiated P4 and senescent ASCs (Supplementary Figure 1), and after three weeks of adipogenic differentiation at P4 and upon senescence (Supplementary Figure 2). Supplementary Figure 3 provides sequencing evidence for a C:T substitution in the *LEP *promoter. Click here for file
