# Supplementary material for: Dynamics of adipogenic promoter DNA methylation during clonal culture of human adipose stem cells to senescence
Source: BMC Cell Biol. 2007 May 29;8:18. doi: 10.1186/1471-2121-8-18 (PMC1892011; doi:10.1186/1471-2121-8-18)
Supplement: Additional file 1 — Supplementary Figures with legends. The data provided show DNA methylation profiles for adipogenic gene promoters in undifferentiated P4 and senescent ASCs (Supplementary Figure 1), and after three weeks of adipogenic differentiation at P4 and upon senescence (Supplementary Figure 2). Supplementary Figure 3 provides sequencing evidence for a C:T substitution in the LEP promoter. [file 1471-2121-8-18-S1.pdf]

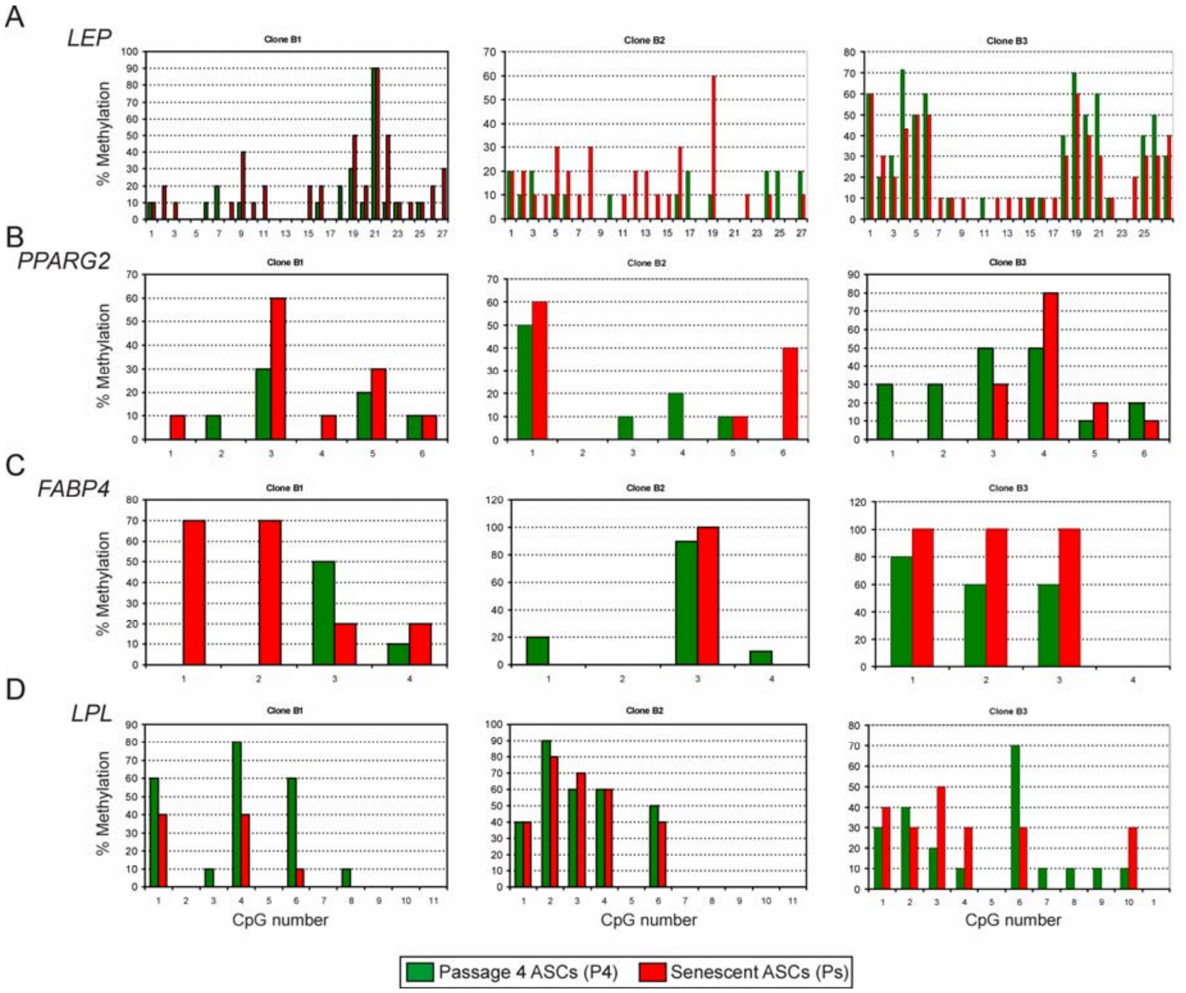

Supplementary Figure 1

DNA methylation of the *LEP*, *PPARG2*, *FABP4* and *LPL* promoters in undifferentiated P4 and senescent (Ps) ASC clones. CpG methylation was analyzed by bisulfite sequencing (Fig. 4A) and data are shown here as percentage of individual methylated CpGs in the (A) *LEP*, (B) *PPARG2*, (C) *FAPB4* and (D) *LPL* promoters for each cell clone.

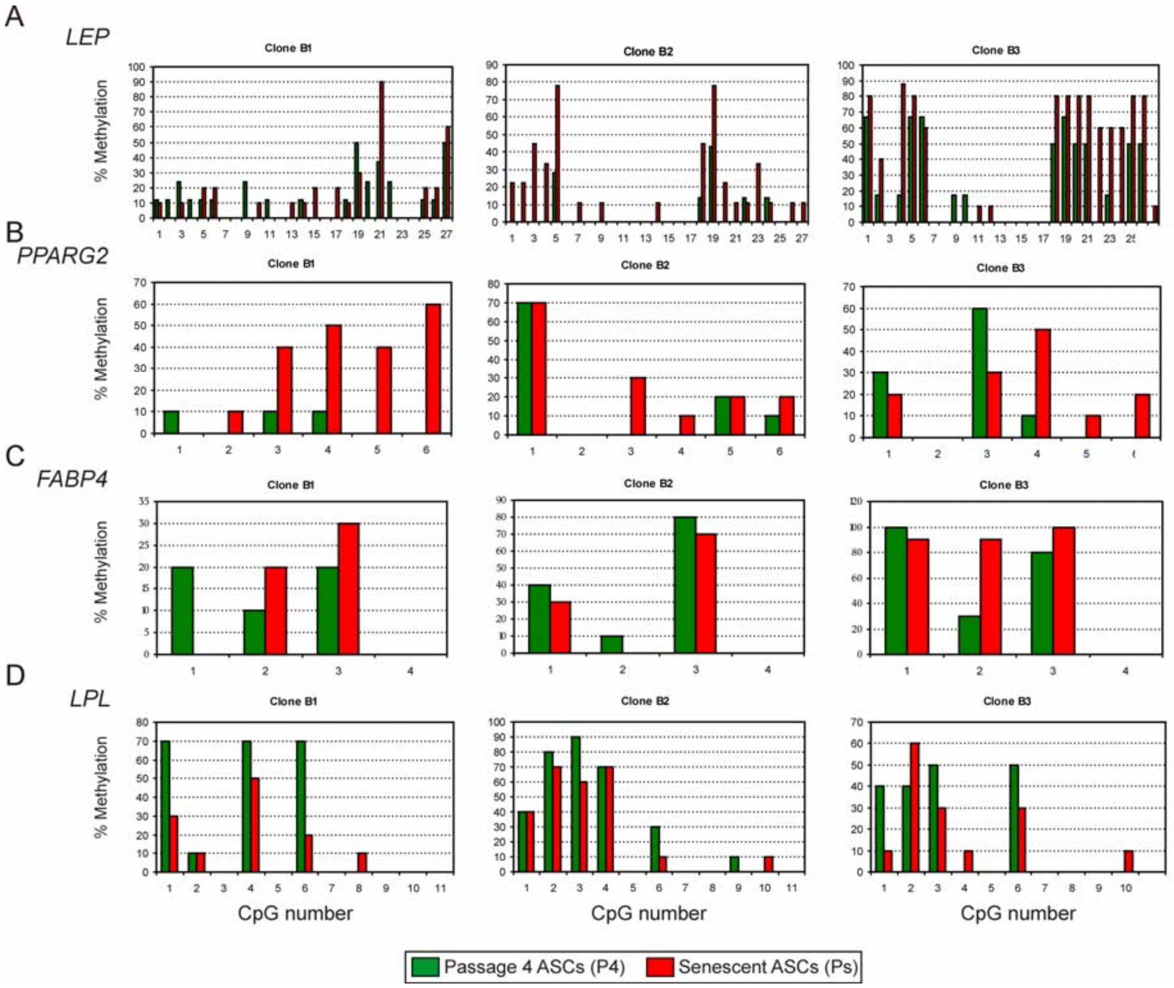

Supplementary Figure 2

DNA methylation of the *LEP*, *PPARG2*, *FABP4* and *LPL* promoters in ASC clones after three weeks of adipogenic stimulation *in vitro*. CpG methylation was determined by bisulfite sequencing (Fig. 4C) and data are shown here as percentage of individual methylated CpGs in the (A) *LEP*, (B) *PPARG2*, (C) *FAPB4* and (D) *LPL* promoters for each cell clone.

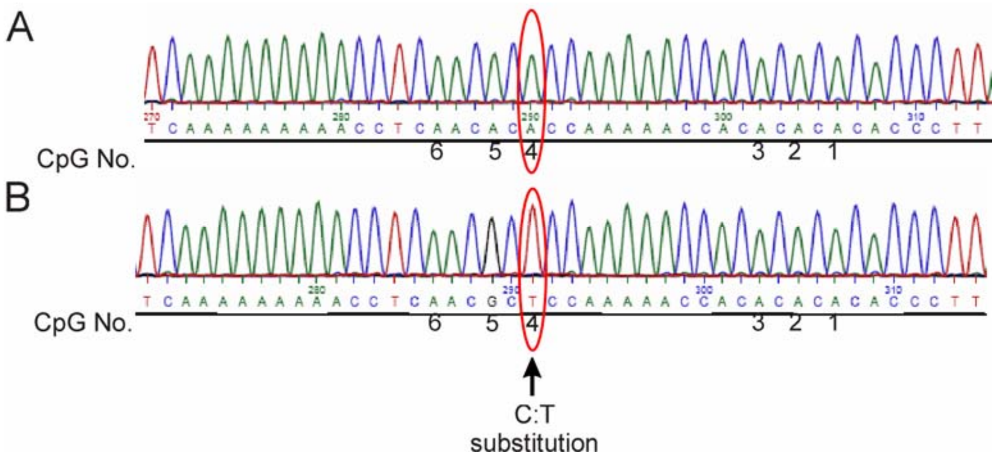

Supplementary Figure 3

Illustration of a C:T substitution detected through bisulfite sequencing. (A) Excerpt of a *LEP* promoter sequence containing 6 potentially methylated cytosines (numbered 1-6), which in this sequence are unmethylated; all read as adenines, or As. (B) In this *LEP* promoter sequence, the cytosine in CpG No. 4 has been substituted for a thymidine (T). Note that the cytosine in CpG. No. 5 is methylated and as such is read as a guanine (G).
